# Psychosoziale Faktoren bei Schmerz und Schmerzbehandlung: Eine Positionsbestimmung

**DOI:** 10.1007/s00482-022-00633-1

**Published:** 2022-03-18

**Authors:** Wolfgang Eich, Anke Diezemann-Prößdorf, Monika Hasenbring, Michael Hüppe, Ulrike Kaiser, Paul Nilges, Jonas Tesarz, Regine Klinger

**Affiliations:** 1 Abteilung Innere Medizin II (Klinik für Allgemeine Innere Medizin und Psychosomatik), Universitätsklinikum Heidelberg, Heidelberg, Im Neuenheimer Feld 410, 69120 Deutschland; 2 Tagesklinik für interdisziplinäre Schmerztherapie, DRK Schmerz-Zentrum Mainz, Auf der Steig 16, 55131 Mainz, Deutschland; 3 Abteilung für Medizinische Psychologie und Medizinische Soziologie, Ruhr-University Bochum, Universitätsstr. 150, 44789 Bochum, Deutschland; 4 Klinik für Anästhesiologie und Intensivmedizin, Universität zu Lübeck, Ratzeburger Allee 160, 23538 Lübeck, Deutschland; 5 Universitäts SchmerzCentrum, Universitätsklinikum Dresden, Fetscherstr. 74, 01307 Dresden, Deutschland; 6 Weiterbildungsstudiengang Psychotherapie, Johannes Gutenberg-Universität Mainz, Lanzelhohl 22, 55128 Mainz, Deutschland; 7 Zentrum für Anästhesiologie und Intensivmedizin, Klinik und Poliklinik für Anästhesiologie, UKE, Martinistr. 52, 20246 Hamburg, Deutschland

**Keywords:** Chronischer Schmerz, Psychosomatik, Schmerzmodelle, Schmerzversorgung, Translation, Chronic pain, Psychosomatics, Models of pain, Pain-care, Translational research

## Abstract

Psychosoziale Faktoren beeinflussen Schmerzerleben und Schmerzgenesung weitreichend, trotzdem ist der Transfer in die klinische Anwendung bisher unzureichend. Mit diesem Beitrag möchte eine Arbeitsgruppe des Arbeitskreises „Psychosoziale Aspekte bei Schmerz“ der Deutschen Schmerzgesellschaft e. V. auf die erhebliche Diskrepanz zwischen bestehender wissenschaftlicher Evidenz zur Bedeutung psychosozialer Faktoren bei der Entstehung chronischer Schmerzstörungen und der Translation dieser Ergebnisse in die Versorgung von Schmerzpatienten aufmerksam machen. Unsere Ziele sind eine stärkere Integration psychologischer und psychosomatischer Expertise in die Schmerzbehandlung und -forschung sowie die Verbesserung der strukturellen und institutionellen Voraussetzungen, um zu einer vermehrten Berücksichtigung psychosozialer Aspekte zu kommen. Nur so können die modernen, integrativen und komplexen Schmerzkonzepte beim Patienten ankommen. Basierend auf diesen grundlegenden Erkenntnissen zur Bedeutung psychosozialer Faktoren bei Schmerz und Schmerzbehandlung sollen Implikationen für den Transfer in die Klinik und die weitere Forschung aufgezeigt werden.

## Abstract (en)

Although psychosocial factors have a profound impact on the experience of pain and pain recovery, the transfer to clinical application has so far been insufficient. With this article, a task force of the special interest group “Psychosocial Aspects of Pain” of the German Pain Society (Deutsche Schmerzgesellschaft e. V.) would like to draw attention to the considerable discrepancy between existing scientific evidence on the importance of psychosocial factors in the development of chronic pain disorders and the translation of these findings into the care of pain patients. Our objective is a stronger integration of psychological and psychosomatic expertise in pain treatment and research, as well as the improvement of structural and institutional conditions, to achieve an increased consideration of psychosocial aspects. In this way, modern, integrative and complex pain concepts can reach the patient. Based on these fundamental findings on the importance of psychosocial factors in pain and pain treatment, implications for the transfer to clinic and further research will be shown.

## Einleitung

Obwohl psychosoziale Faktoren Schmerzerleben und Schmerzgenesung weitreichend beeinflussen, ist der Transfer in die klinische Anwendung bisher unzureichend. Mit diesem Beitrag möchte eine Arbeitsgruppe des Arbeitskreises „Psychosoziale Aspekte bei Schmerz“ der Deutschen Schmerzgesellschaft e. V. auf die erhebliche Diskrepanz zwischen bestehender wissenschaftlicher Evidenz zur Bedeutung psychosozialer Faktoren bei der Entstehung chronischer Schmerzstörungen und der Translation dieser Ergebnisse in die Versorgung von Schmerzpatienten aufmerksam machen. Unsere Ziele sind eine stärkere Integration psychologischer und psychosomatischer Expertise in die Schmerzbehandlung und -forschung sowie die Verbesserung der strukturellen und institutionellen Voraussetzungen, um zu einer vermehrten Berücksichtigung psychosozialer Aspekte zu kommen. Nur so können die modernen, integrativen und komplexen Schmerzkonzepte beim Patienten ankommen.

## Schmerzforschung

*Fortschritte der Schmerzforschung und -behandlung: Schmerz ist immer somatisch und psychisch zugleich*.

Die moderne Schmerzforschung orientiert sich an einem biopsychosozialen Krankheitsmodell: die gleichzeitige Berücksichtigung somatischer, psychischer und sozialer Faktoren und Mechanismen bei der *Entstehung, Aufrechterhaltung und Behandlung* von Schmerzzuständen ist erforderlich, um die Komplexität der Schmerzerkrankung und das Erleben auf Seiten der Patienten zu verstehen [29, 32, 43]. Die Deutsche Schmerzgesellschaft definierte dies als Ausgangspunkt ihrer Forschungsagenda [18] und sieht die Schmerzforschung der Zukunft als interdisziplinär, translational, transparent und am biopsychosozialen Krankheitsmodell ausgerichtet. Biopsychosoziale Faktoren sollen von der Grundlagenforschung bis hin zur Versorgungsforschung des Schmerzes methodisch und klinisch berücksichtigt werden.

Psychologische und soziale Faktoren haben sich in zahlreichen klinischen Studien als wichtige Schaltstellen erwiesen, die den Übergang vom akuten zum chronischen Schmerz charakterisieren, zur Entstehung und Aufrechterhaltung der Schmerzen beitragen und ggfs. auch deren räumliche Ausbreitung begünstigen [87]. Psychosoziale Faktoren wurden als „Risikofaktoren“ bei postoperative Schmerzen, akuten Rückenschmerzen, Kopfschmerzen, Bauchschmerzen und anderen chronischen Schmerzzuständen untersucht (z. B. [34, 50–52]). Identifiziert wurden dabei Katastrophisierungsgedanken, Ängste, depressive Symptome, Kontrollverlust, Aufmerksamkeit auf den Schmerz und Unsicherheit über den Verlauf der Schmerzen sowie Lern- und Gedächtnisprozesse (z. B. [27, 46, 77]).

Das enge Zusammenspiel zwischen neurobiologischen und psychologischen Faktoren ist mittlerweile sehr differenziert erforscht worden: Schmerz wird durch neurobiologische und psychologische Faktoren moduliert. Psychische Faktoren modulieren die Wahrnehmung und Verarbeitung von Schmerzen, unabhängig davon, ob es sich um akute oder chronische Schmerzen handelt. Zusammen mit körperlichen Faktoren führen diese durch Lernen und neurobiologische Prozesse zu Veränderungen im Nervensystem, der sogenannten Neuroplastizität (z. B. [27]). Diese können reversibel sein (z. B. [49]), aber auch unter noch nicht identifizierten Bedingungen irreversibel werden [73].

Die immer wieder erwiesene Komplexität des Zusammenwirkens psychischer und somatischer Faktoren wird auch aus Befunden der *Analgetikaforschung* deutlich. So konnte gezeigt werden, dass sich soziale Ausgrenzung in einer veränderten Aktivität von Hirnstrukturen auswirkt, auf die auch Nicht-Opioidanalgetika Einfluss nehmen (z. B. der dorsale anteriore cinguläre Kortex: dACC). Die Präparateeinnahme führt zu Schmerzreduktion und zur Änderung der emotionalen Reagibilität [71].

Der gegenwärtige Forschungsstand zur *Modulation von Schmerzinformation* (aus der Peripherie oder aus dem ZNS) *durch psychosoziale Faktoren* ist sehr differenziert und komplex (z. B. [6, 10, 13, 16, 21, 23, 26, 27, 58]).

In Abhängigkeit von der motivationalen und aufmerksamkeitsbezogenen Verfassung eines Menschen kann der gleiche nozizeptive Reiz in einem Kontext als stark schmerzhaft erlebt werden, in einem anderen Kontext als lediglich gering schmerzhaft [29]. Dies geschieht beispielsweise über die absteigenden Schmerzbahnen, die die Übertragung von Schmerzsignalen im Hinterhorn des Rückenmarks modulieren. Das Ausmaß dieser Modulation unterliegt ganz wesentlich dem Einfluss psychosozialer Faktoren. Als psychosoziale Faktoren sind bspw. die Aufmerksamkeit und Erwartungshaltung, aber auch Kognitionen wie die individuelle Schmerzbewertung, Emotionen und die kognitive Reflexion sozialer Kontexte zu nennen [53]. Diese Mechanismen wirken aber nicht nur auf komplexe Weise auf die Schmerzempfindung selber, sondern interagieren untereinander und bedingen auf diese komplexe Art und Weise eine höchst individuelle und situative Schmerzempfindung.

Es besteht ebenso ein enger Zusammenhang zwischen der *emotionalen Stimmung* und der subjektiven Schmerzbewertung. Gedanken, Evaluationen und Erwartungen können Stimmungen auslösen und den Anstoß für bestimmte Verhaltensweisen geben und dadurch wiederum die Umwelt beeinflussen – und umgekehrt. So wird ein erwarteter oder vertrauter Schmerz als weniger schmerzhaft erlebt als ein unerwarteter, neuer Schmerz [81]. Nieto [63] konnte zeigen, dass Schmerz wesentlich und günstig von den individuell verfügbaren Bewältigungsmechanismen beeinflusst wird. Eine weitere Rolle spielen Ängste und Depressionen [33], denn sie erhöhen die Morbidität und führen letztlich auch zu hohen sozioökonomischen Kosten [7].

Zu den psychologischen Risikofaktoren zählt weiterhin der *verhaltensbezogene Umgang* mit Schmerzen [34]. Sowohl das frühzeitige Vermeiden körperlicher Aktivitäten (z. B. sportliche Betätigung, aber auch Gehen, Treppensteigen im Alltag) oder sozialer Anforderungen (z. B. das Annehmen von Einladungen von Freunden) als auch das ausgeprägte Durchhalten aller Aktivitäten selbst bei starken Schmerzen tragen zur Aufrechterhaltung der Schmerzen bei, vermutlich vermittelt über Minder- oder Überlastung körperlicher Strukturen, wie etwa von Muskulatur, Bändern, Bandscheiben, Faszien und Gelenkstrukturen im Falle chronischer Rücken- und Beinschmerzen [36] im Sinne einer erhöhten Reagibilität des Nervensystems. Beide Verhaltenstendenzen, so eine Metaanalyse aus dem Jahre 2012 [1], gehen offenbar mit einem Mehr an Schmerzintensität einher. Es wird angenommen, dass die psychologischen Verhaltenstendenzen ihrerseits über operante Lernprozesse aufrechterhalten werden [85] und so zu körperlicher In- oder Überaktivität beitragen. Der Zusammenhang zwischen Rückenschmerzen mit sehr geringer körperlicher Aktivität, aber auch mit hoher Aktivität konnte z. B. von Heneweer et al. 2009 [39] im Rahmen einer groß angelegten epidemiologischen Studie an mehreren Tausend Probanden überzeugend gezeigt werden. Erste Akzelerometer-basierte Studien unterstützen diese Zusammenhänge bei Patienten mit Rückenschmerzen nach einer Bandscheiben-OP, wobei die psychologischen Verhaltenstendenzen des Vermeidens wie auch Durchhaltens mit sehr geringen oder sehr hohen Aktivitätsleveln im Alltag einhergingen [35, 68]. Leeuw et al. [50] zeigen in einem Review klare Evidenz, dass „fear avoidance beliefs“ der Patientinnen und Patienten einen robusten Risikofaktor für den Übergang vom akuten zum chronischen Schmerz, einen essenziellen Prädiktor für anhaltende „disability“ und einen validen Prädiktor für den Krankheitsverlauf und Therapieergebnis darstellen.

Eine durch entsprechende Verhaltensweisen getriggerte körperliche Minder- oder Überaktivität könnte mit Ursache dafür sein, dass die physiotherapeutischen Anteile multimodaler Therapieansätze häufig nur einen sehr moderaten Erfolg aufweisen. Qlugbade et al. [70] fanden Angst als Mediator zwischen Schmerz und einem „guarded movement“. Der Fokus der Therapie sollte hier weniger auf viel Bewegung als auf kognitiv-emotionale Aspekte und den Aufbau von Zutrauen zur Bewegung gelegt werden.

Erste Schritte, die psychologischen Tendenzen des Meidens oder Durchhaltens bei der Durchführung direkt aktivitätssteigernder Programme zu berücksichtigen, scheinen hier die Erfolgsrate erhöhen zu können (siehe den Forschungsverbund „Medicine in Spine Exercise – MiSpEx“, www. Ranrücken.de). Diese Befunde zeigen einen erhöhten Forschungsbedarf in diesem Bereich an.

Eine weiterhin wichtige und häufig vernachlässigte Rolle kommt psychischen *Traumatisierungen* für die Entstehung und Aufrechterhaltung von Schmerzen zu [17, 37, 38, 60, 75, 78]. Folgt man den empirischen Befunden, dann stellen psychische Traumatisierungen ein erhebliches Risiko für die Entwicklung chronischer Schmerzen dar, weil sie auf ähnliche zentralnervöse Mechanismen wirken („common pathway model“) und eine vorhandene Schmerzsymptomatik nach oben modulieren [87]. Dies gilt auch für frühkindliche Traumatisierungen [59]. Die gemeinsamen Mechanismen der Verarbeitung von Angst, Depression und psychologischen Traumata bewirken zentral auch eine Senkung der Schmerzschwellen und verändern damit das Schmerzempfinden relevant [79].

Einige der aufgeführten psychologischen Faktoren wurden in letzter Zeit unmittelbar experimenteller Forschung zugänglich gemacht. So liefert seit einigen Jahren die *Placeboforschung* zahlreiche experimentelle Befunde zur Schmerzmodulation über kognitive und affektive Faktoren [5, 8, 24, 57]. Mit der experimentellen Forschung zum Phänomen des Placebo- und auch Noceboeffekts lassen sich die äußerst komplexen psychobiologischen Vorgänge aus Lern- und Erwartungsprozessen in ihrer Wechselwirkung mit Emotionen und Kognitionen und mit neurobiologischen Prozessen des Gehirn darstellen [48]. Die Placeboforschung ist ein wichtiges und wegweisendes Modell, um Phänomene wie Erwartungen über Krankheitsverläufe, Behandlungserwartungen, Lernmechanismen und Kontextfaktoren zur Schmerzverstärkung und Schmerzhemmung zu erforschen [9, 11, 15, 25, 66, 69]. Die neuen Kenntnisse werden bereits unter dem Aspekt der klinischen Anwendung diskutiert (z. B. [20, 47]). Durch systematische Koppelung von wirkungsvollen psychologischen Mechanismen an wirkstofffreie Substanzen – sozusagen als Träger – entsteht eine neue Qualität psychotherapeutischer Interventionen für akuten und chronischen Schmerz. Diese Koppelung kann auch an potente Medikamente erfolgen, die so ihre Wirksamkeit steigern können. Allerdings ist noch ein großer Forschungsaufwand notwendig, um praxistaugliche Interventionen zu formulieren und die notwenige Struktur der Kooperation zwischen psychotherapeutischen und somatischen Interventionen zu entwickeln [45].

Auch zum *Zusammenhang zwischen körperlicher Aktivität und Schmerz* wurde mit dem Konzept der bewegungsinduzierten Hypoalgesie (engl. „exercise-induced hypoalgesia“ [EIH]) ein experimentelles Paradigma entwickelt, mit dem in zahlreichen Studien gesunde Probanden wie Patienten mit chronischen Schmerzen untersucht wurden (s. Review von Rice et al. [72]). Einer der zentralen Befunde besagt, dass es bei gesunden Probanden unter körperlicher Aktivität (z. B. 15 min aerobe Belastung) zu einer Erhöhung der Schmerzschwellen kommt, während dieser Effekt bei hochchronifizierten Schmerzpopulationen ausbleibt oder gar in einer erhöhten Schmerzsensitivität mündet. Erste kürzlich durchgeführte Studien zeigten darüber hinaus wiederum Wechselwirkungen zwischen dem EIH-Effekt und der experimentell manipulierten Erwartungshaltung über den Effekt von sportlicher Aktivität [84] bzw. Tendenzen zu kognitiver Inhibitionsfähigkeit [30]. Beide Ansätze, die experimentelle Placebo- wie auch die EIH-Forschung, können so die bislang primär in klinischen Studien gewonnenen Zusammenhänge zwischen zentralen psychologischen Faktoren und dem Schmerzerleben untermauern und zudem potenzielle Mechanismen auf Ursache-Wirkungs-Zusammenhänge überprüfen.

Diese Perspektive psychosozialer Faktoren bei der Entstehung, Aufrechterhaltung und auch räumlichen Ausbreitung von Schmerzen eröffnet Möglichkeiten psychologischer und psychosomatischer Diagnostik und Interventionen in der Schmerztherapie. In ihrer Forschungsagenda „Schmerz“ [18] formuliert die Deutsche Schmerzgesellschaft die stärkere Betrachtung schmerzmodulierender Faktoren in der zukünftigen Schmerzforschung als wesentliches Ziel. Biopsychosoziale Interaktionen und Mechanismen müssen sowohl in der Grundlagenforschung als auch in der klinischen Forschung und der Versorgungsforschung berücksichtigt werden. Die Bedeutung von biopsychosozialen und somatopsychischen Interaktionen spiegelt sich auch in der bei chronischen Schmerzpatienten wiederholt berichteten Übersterblichkeit wider [76, 80], welche auf die komplexen Wechselwirkungen zwischen Schmerz und somatischen und psychologischen Komorbiditäten zurückgeführt wird [76, 80]. Die Umsetzung in die Versorgung erfordert nicht nur die Stärkung entsprechender Schnittstellen in der Zusammenarbeit der beteiligten Fachdisziplinen (Medizin, Psychologie, Physiotherapie und Pflege), sondern wesentlich die Etablierung interdisziplinärer Netzwerke, die sich als Team mit gemeinsamem Ziel verstehen und bereits frühzeitig in der Versorgung sowohl in Diagnostik als auch therapeutischen Interventionen ansetzen. Die Umsetzung setzt aufseiten der Behandelnden spezifische Qualifikationen voraus (Medizin: spezielle Schmerztherapie; Psychologie: spezielle Schmerzpsychotherapie; Physiotherapie: spezielle Schmerzphysiotherapie, Pflege: spezielle pflegerische Schmerzweiterbildung).

## Interdisziplinäre multimodale Schmerzbehandlung

*Aufgrund der Wechselwirkung psychologischer und somatischer Mechanismen des Schmerzes ist eine Schmerzbehandlung dann besonders erfolgreich, wenn sie interdisziplinär und multimodal angelegt ist*.

In den letzten Jahrzehnten hat sich die Auffassung über die Entstehung und das Verständnis chronischer Schmerzen als ein Lernprozess auf psychosozialer und neuronaler Ebene durchgesetzt. Psychosoziale Aspekte bilden in der interdisziplinären Diagnostik (14, 74, 86) und Therapie chronischer Schmerzformen eine wesentliche Säule, die auf die oben genannten wissenschaftlichen Erkenntnisse aufbaut und diese integriert.

National und international wurden neue, komplexe und meist interdisziplinär angelegte Behandlungskonzepte insbesondere für chronische Schmerzformen entwickelt [31, 56]. In der ICD-11 ist „primärer chronischer Schmerz“ eine eigenständige Diagnose (MG30.xx) außerhalb des F‑Kapitels für psychische Störungen. Weitere sechs Diagnosen wurden für sekundären chronischen Schmerz eingeführt [64, 83].

Die Diagnosen entsprechen einem biopsychosozialen Schmerzverständnis [22] und begründen die Indikation für eine interdisziplinäre multimodale Schmerztherapie (IMST) bei chronischen Schmerzen [65], in der somatische sowie psychotherapeutische Verfahren gleichzeitig mit individualisierten Schwerpunkten angewandt werden [3, 61, 82]. Gefordert wird die Zusammenarbeit von in der Schmerzbehandlung spezialisierten Ärzten (Anästhesisten, Orthopäden, Neurologen, Psychosomatischen Medizinern etc.), Bewegungstherapeuten (Moto‑, Sport‑, Ergo- und Physiotherapeuten), Psychotherapeuten sowie Pflegepersonal und Kotherapeuten (Tanztherapeuten, Sozialtherapeuten etc.). Im Vordergrund stehen die Wiederherstellung der körperlichen und psychischen Leistungsfähigkeit [2] und damit die Verminderung der Beeinträchtigung im Alltag, ein verbesserter Umgang mit den Beschwerden, die Verringerung der psychischen Belastung (z. B. der Stimmung) und eine Reintegration in das Berufs- und Sozialleben.

Diese interdisziplinär-multimodalen Programme haben sich bisher in unterschiedlichen Settings und bei verschiedenen chronischen Störungsbereichen als wirksam erwiesen und waren in einigen Studien unimodalen Therapieansätzen überlegen [28, 42, 69]. Allerdings setzen sie die Kooperation innerhalb und zwischen Fachdisziplinen voraus, und zwar nicht nur als additives Nebeneinander, sondern als ein Netzwerk mit engen räumlichen und zeitlichen Beziehungen und einem übergeordneten Behandlungskonzept. Interdisziplinarität in diesem Sinne bedeutet Behandlung durch ein multidisziplinäres Team, das bei der Beurteilung und Behandlung mit einem gemeinsamen biopsychosozialen Modell und Zielen zusammenarbeitet, mit regelmäßigen Teambesprechungen, Vereinbarungen zur Diagnose, zu therapeutischen Zielen und zu Plänen für die Behandlung und Überprüfung [40]. Diese Behandlungsform ist hinsichtlich Intensität und mittelfristiger Ersparnis für die Gesellschaft erfolgreich [62], gleichzeitig aber auch hinsichtlich organisatorischem Aufwand und unmittelbaren Kosten anspruchsvoll. Auch wenn in Deutschland bereits zahlreiche Einrichtungen entstanden sind, ist die Situation von einer flächendeckend ausreichenden und sektorenübergreifenden und interdisziplinären Versorgung weit entfernt [19]. Auch werden bei den Abrechnungsmöglichkeiten Teamleistungen nicht angemessen vergütet. Sektorenübergreifende, durchlässigere und flexiblere Versorgungsmöglichkeiten werden dadurch erheblich erschwert. Die „Wirtschaftlichkeitsprüfungen“ durch den Medizinischen Dienst der Krankenkassen erschweren zusätzlich diesen Sachverhalt [55].

## Entwicklungen, Perspektiven und Ziele für die interdisziplinäre Diagnostik und Schmerzbehandlung

Die Fortschritte der Schmerzforschung basieren zu einem beträchtlichen Teil auf psychologischen und neurokognitiven Modellen und Kenntnissen. Der Transfer dieser Erkenntnisse in die Praxis der schmerztherapeutischen Behandlung ist allerdings erst in Ansätzen gelungen, erfährt aber durchaus derzeit einige Berücksichtigung. Auch die psychologische und psychosomatische Expertise bei drittmittelgeförderter Schmerzforschung ist nach wie vor unterrepräsentiert.

Die Deutsche Schmerzgesellschaft hat verschiedene Projekte angestoßen, um diese Defizite vor allem in Bezug auf die interprofessionelle Zusammenarbeit zu beheben. Mit finanzieller Förderung durch den Innovationsfonds wurden die Projekte PAIN 2020 (www.pain2020.de) und seit kurzem PAIN 2.0 (www.schmerzgesellschaft.de/topnavi/forschung-und-foerderung/forschunsgfoerderung-1) angestoßen. In ausgewählten Praxen und Kliniken wird Patienten mit dem Risiko einer Schmerzchronifizierung möglichst frühzeitig eine interdisziplinäre Diagnostik und Behandlung angeboten. Dabei werden insbesondere psychosoziale Risikofaktoren für eine Chronifizierung von Schmerzen sowohl in der Gestaltung von diagnostischen Angeboten als auch von therapeutischen Interventionen früher als bisher üblich in die Betreuung von Betroffenen integriert [41, 44]). Diese Bestrebungen verfolgen dabei Leitlinien, die frühzeitige interdisziplinäre Interventionen und Diagnostik dringend empfehlen (bspw. [12]).

Die interdisziplinäre Grundeinstellung, aber auch die frühzeitige Versorgung mit psychotherapeutischen Angeboten, wird darüber hinaus auch in dem Innovationsfondprojekt „POET-Pain“ umgesetzt, das ebenfalls unter Konsortialführung der Deutschen Schmerzgesellschaft e. V. durchgeführt wird. In mehreren Universitätskliniken wird ein „Transitional Pain Service“ eingerichtet, der aus einem festen Team aus den Berufsgruppen Medizin, Psychologie, Physiotherapie und Pflege besteht. Ziel der interdisziplinären Behandlung ist die Verhinderung der Entwicklung persistierender postoperativer Schmerzen bei Patienten mit erhöhtem Risiko für eine chronische Schmerzentwicklung nach Operationen.

Für eine qualitativ hochwertige Versorgung spielt gerade im Rahmen der IMST die Qualifikation der Professionen eine große Rolle. Für alle beteiligten Fachdisziplinen der IMST besteht Konsens, dass die Basisqualifikation im jeweiligen Beruf für eine angemessene Versorgung von Menschen mit Schmerz nicht ausreicht. Medizin, Psychologie, Physiotherapie und Pflege haben Weiter- bzw. Fortbildungen zur Verbesserung von Struktur- und Prozessqualität entwickelt.

Aufgrund der zunehmenden Kommerzialisierung und der Tendenz einiger Leistungsanbieter, Erlöse zu maximieren und Personalkosten zu minimieren, wurden die Kriterien für Struktur- und Prozessmerkmale zunehmend präzisiert und angehoben [4]. So ist seit 2011 u. a. die Approbation zumindest eines Psychologischen Psychotherapeuten im Team Voraussetzung für die IMST [54].

### Forderungen für die ambulante Schmerztherapie

*Es besteht eine erhebliche Diskrepanz zwischen den am Schmerz beteiligten Faktoren (psychosozial und somatisch) und deren Berücksichtigung in der ambulanten Behandlung*.

Eine Verankerung psychosozialer Versorgungsstrukturen im ambulanten Bereich ist vor dem Hintergrund der hohen Prävalenzen von Schmerzstörungen dringend erforderlich. Dies beinhaltet:

1. Die frühzeitige Identifikation von Patientinnen und Patienten mit psychosozialen Risikofaktoren durch Haus- und Fachärzte. Trotz allem praktischen Wissen erhalten hier die zur Chronifizierung neigenden Patienten oft erst in (zu)späten Chronifizierungsphasen eine adäquate Diagnostik und Therapie. Hier sollten alle Beteiligten enger und intensiver zusammenarbeiten: Haus- und auch Fachärzte, aber auch Physiotherapeuten sollten Möglichkeiten bekommen, zur Chronifizierung neigende Patienten rasch einer interdisziplinären, multimodalen Schmerztherapie (IMST) mit schmerzpsychologischer Expertise zuzuführen.
2. Die Begleitung von Patientinnen und Patienten innerhalb eines Netzwerks an die richtigen Anlaufstellen, und die Koordination der indizierten multimodalen Behandlungselemente. Hier gibt es Verbesserungsbedarf im Versorgungssystem. Es gibt einerseits hochspezialisierte Einrichtungen für Schmerzbehandlung, ambulante Schmerzsprechstunden (z. B. Kopfschmerz, Schmerz allgemein) an den Universitätskliniken, ambulante und tagesklinische Schmerzzentren, stationäre Schmerzeinrichtungen, andererseits im niedergelassenen Bereich Schmerztherapeuten, Psychosomatiker und Psychologische Psychotherapeuten. Eine angemessene Vernetzung all dieser schmerztherapeutischen Angebote findet aber noch nicht in ausreichendem Maße statt. Viele Patienten mit chronischen Schmerzen finden sich innerhalb dieser verschiedenen Behandlungsstrukturen nur schwer zurecht. Schmerzbehandler bzw. -behandlungszentren arbeiten so oft „nebeneinander her“. Somit ist es eine der aktuell wichtigsten Aufgaben, die bestehenden Behandlungsmöglichkeiten für den einzelnen Patienten aufzuzeigen und diese so zu vernetzen, dass angebotenen Therapiemöglichkeiten erfolgsversprechend ausgeschöpft und koordiniert werden können.
3. Eine adäquate ambulante, niederschwellige Nachbetreuung nach spezifischen multimodalen Therapieinterventionen zur dauerhaften Stabilisierung chronischer Schmerzpatienten.

Die Ad-hoc-Kommission der Deutschen Schmerzgesellschaft hat Empfehlungen zu Struktur- und zu Prozessparametern erarbeitet, die flächendeckend – auch im ambulanten Feld – umgesetzt werden sollten [67], um die betroffenen Patienten nach den Qualitätserfordernissen einer adäquaten IMST zu behandeln. Darüber hinaus werden derzeit Modellprojekte und Vertragsentwürfe zur Umsetzung interdisziplinärer ambulanter Schmerztherapie entwickelt und diskutiert.

In der ambulanten Versorgung mangelt es derzeit noch an schmerzpsychotherapeutischen Behandlungsmöglichkeiten und im Alltag praktikablen Kooperationsformen. Ursache dafür ist weniger eine fehlende Bereitschaft zur Zusammenarbeit. Vielmehr führt die für Deutschland geltende generelle psychotherapeutische Unterversorgung auch für die Schmerztherapie zu langen Wartezeiten und damit zu erheblichen Problemen in der ambulanten Patientenversorgung.

### Forderungen für die teilstationäre und stationäre Schmerztherapie

*In allen Krankenhäusern, die ambulante, teilstationäre und/oder stationäre Behandlungen für Patienten mit chronischen Schmerzen anbieten, müssen Patienten einen Zugang zu psychotherapeutischen Spezialisten für die Schmerzbehandlung haben*.

In ausgewählten größeren Allgemeinkrankenhäusern und Krankenhäusern der Maximalversorgung sollten organisatorische Einheiten für die spezialisierte Schmerzbehandlung eingerichtet werden, in denen auch interdisziplinäre multimodale Therapieverfahren für chronische Schmerzpatienten angeboten werden. Bei der multimodalen Schmerzbehandlung bilden psychologische bzw. psychosomatische Verfahren einen wesentlichen Baustein (siehe [3]).

Die Organisationsform solcher Behandlungseinheiten hat dem interdisziplinären und multiprofessionellen Charakter der multimodalen Behandlung Rechnung zu tragen. Spezialisten aus den drei Bereichen Algesiologie, Psychosomatik/Psychologie und Bewegungs‑/Ergotherapie sind daran zu beteiligen. Diese können je nach lokaler Gegebenheit von verschiedenen Abteilungen/Kliniken gestellt werden (Anästhesie, Innere Medizin, Neurologie, Psychosomatische Medizin, Psychiatrie, medizinische und klinische Psychologie, Physikalische Medizin und Orthopädie/Rheumatologie).

Eine spezialisierte Schmerzbehandlung sollte insbesondere für bisher vernachlässigte Gruppen von Patienten vorgesehen werden, wie z. B. chronisch Kranke, ältere Patienten, Kinder und Jugendliche sowie Patienten mit Migrationshintergrund.

### Wichtige Themen für die zukünftige Schmerzforschung

*In allen Forschungsvorhaben zum chronischen Schmerz (insbesondere auch in klinischen, somatisch/physiologischen Forschungen) müssen psychosoziale Aspekte integriert werden, da sonst die Aussagekraft erheblich eingeschränkt wird*.

Einhergehend mit der Forschungsagenda der Deutschen Schmerzgesellschaft (vgl. [18]) sollten die Herausforderungen der Schmerzforschung in der Zukunft vor allem auf den folgenden Aspekten liegen:

- Erwartungen der Patienten stärken in Richtung Selbsteffizienz, Selbstmanagement und Aktivität bei Schmerz
- Erwartungen und finanzielle Anreize in der Medizin mehr auf Stärkung eigener Ressourcen und Schmerzbewältigung bei den Patienten richten: Erwartung und jahrelange Konditionierung des Patienten verändern und innovative Konzepte zugänglich machen
- Adäquate Informationsvermittlung steuern vs. Informationsflut eindämmen
- Mehr Zeit für interdisziplinäre Zusammenarbeit und für den Kontakt mit Patienten schaffen
- Adäquate Anwendung des Wissens/therapeutischer Möglichkeiten
- Interdisziplinäre und interprofessionelle Kommunikation zwischen Forschern und Anwendern fördern und stärken
- Stärkung der psychologischen und psychosomatischen Grundlagenforschung und Modellbildung zu Schmerz unter Berücksichtigung von emotionaler Befindlichkeit, Traumatisierungen, verhaltensbezogenem Umgang mit Schmerz, Aktivitäts- und Bewegungslevel sowie der Rolle von Angst und Depression sowie die Entwicklung neuer therapeutischer Ansätze.
